# Supplementary material for: Unraveling hidden interactions in complex systems with deep learning
Source: Sci Rep. 2021 Jun 17;11:12804. doi: 10.1038/s41598-021-91878-w (PMC8211832; doi:10.1038/s41598-021-91878-w)
Supplement: Supplementary file 1 — Supplementary Information. [file 41598_2021_91878_MOESM1_ESM.pdf]

Supplementary Materials for ”Unraveling hidden interactions in complex systems with deep learning”

1 Supplementary Methods : Dataset details

Cellular automata We employed the cellular automata (CA) model based on the rule of Conway's life game [1]. As described in the main manuscript, CA takes place on regular grids with each cell on the grid altering its cell state at each time step according to a specific set of rules. These rules are often notated as $B3/S23$, which means a dead cell regenerates with three neighboring live cells, while a live cell stays alive with two or three neighboring live cells. In this study, we used a 14×14 grid with uniformly random initial cell states and updated an inner square grid of 12×12 to avoid the periodic boundary problem. We simulated 1,000 sets of samples for demonstration, comprising 800 samples for training and 200 samples for testing. We report that fewer samples such as 500 or 300 also resulted in a perfectly trained model with 100% test accuracy. Each target cell along with its eight neighbor cells yields a total of $2^9 = 512$ possible microstates. We located a 3×3 microstate template at a random position on the grid cell, initialized to random cell states, and produced AgentNet output. If the model correctly learned the transition rule, it would result in theoretical output assigned by the transition rule of CA regardless of its position and other irrelevant cell states.

From the perspective of AgentNet, CA is a binary classification problem, i.e. whether each cell becomes alive or dead at the next time step. AgentNet for CA receives three state variables:

positions $\mathbf{x}^t$ and $\mathbf{y}^t$, and cell state $\mathbf{c}^t$.The output here is a list of expected probabilities that each cell becomes alive. We use the binary cross entropy loss function between the AgentNet output and the ground-truth label as

$$L_{BCE}(\mathbf{s}) = \sum_i y_i \log c_i + (1 - y_i) \log (1 - c_i), \tag{1}$$

where c_i is the predicted ith cell state of output $\mathbf{s}$, and y_i is the corresponding true label of the ith cell.

Vicsek model The Vicsek model (VM) [2] assumes that flocking occurs due to velocity alignment with neighbors. Among the many variants, we implemented the simplest model with alignment terms and positional Gaussian noise. (Note that this differs from the originally proposed model [2], which used angular Gaussian noise instead.) In VM, the ith agent interacts with the jth agent if the distance between the two agents, $r(s_i^t, s_j^t)$, is smaller than a certain range, r_c, and the absolute value of the angle between the heading direction of the ith agent and the position of the jth agent, $\theta(s_i^t, s_j^t)$, is smaller than a certain angle, θ_c. This interaction range models the sight range limit of living organisms, resulting in a circular sector form. In VM, the ith agent averages the velocity among its interacting neighbors R_i and adds Gaussian noise $\mathcal{N}(0, \sigma)$ to compute its velocity $\mathbf{v}_i^{t+1}$, described as follows:

$$\mathbf{v}_i^{t+1} = (\mathbf{v}_i^t + \sum_{a_j \in R_i} \mathbf{v}_j^t)/(|R_i| + 1) + \mathcal{N}(0, \sigma).$$

This formula is equivalent to $(\sum_{R_i^*} \mathbf{v}_i)/|R_i^*| + \mathcal{N}(0, \sigma)$ where $R_i^* = R_i \cup a_i$.

AgentNet for VM aims to predict the position of the next time step, which is a sum of the current position and calculated velocity. We simulated 2,000 sets of samples for demonstration, comprising 1,600 samples for training and 400 samples for testing. In our simulation, $r_c = 1$, $\theta_c = 120°$, and standard deviation of noise $\sigma = 0.2$.

The model infers the parameters of two 1D Gaussian distributions, which is means (μ^x, μ^y) and σ^x, σ^y. We calcualte the sum of the NLL loss function for Gaussian distribution to train the AgentNet for VM as

$$L_{NLL}(\mathbf{s}) = \sum_q \sum_i -\frac{1}{2}\log(2\sigma_i^q) + \frac{(\mathbf{y}_i^q - \mu_i^q)^2}{2(\sigma_i^q)^2}, \tag{2}$$

where μ_i^q and σ_i^q are the predicted statistics of variable q of the ith agent, and $\mathbf{y}_i^q$ is the corresponding label of the ith cell. In the VM case, $q = \{x, y\}$.

Active Ornstein–Uhlenbeck particle For the AOUP dataset, we simulated 8,000 sets of training data and label pairs of 100 particles. All particles were uniformly spread on a circle of radius 5, and initial speeds were sampled from the uniform distribution $\mathcal{U}(0, 0.05)$. We implemented the Euler–Maruyama [3] method with timestep $dt = 0.01$ to numerically simulate the AOUP trajectories. The data and label points were further subsampled from the simulated trajectory with a frequency of 10 Hz, which means every 1 out of 10 subsequent data points were chosen. In terms of real-time, our model receives 0.8 s of data observation time and then predicts the following 1.2 s of trajectory. The following constants were adopted for simulation: $\gamma = 1$, $\tau = 0.5$, $k = 0.1$, $D_a = 0.02$, and

$T = 0.2$. We have found that our model is robust against change in system constants, showing similar performance with different constant values.

We attached a constant vector R to the attention vector to open the possibility that interaction function h_{pair} depends on the global variable R. (In case of AOUPs, this is the case since R affects interaction potential $\mathbf{F}^{int}$.) Differing from the VM case, AgentNet for AOUP yields a total of 8 parameters for each agent, namely means and variances for positions $\mathbf{x}^{t+1}$, $\mathbf{y}^{t+1}$ and velocities ${\mathbf{v_x}}^{t+1}$, ${\mathbf{v_y}}^{t+1}$. This is necessary because input and output state variables should be the same in order to iteratively sample from the predicted distribution and feed it into the prediction at the next step.

We employed teacher forcing [4] to train the LSTM-based AgentNet, which is a technique that feeds ground-truth labels into subsequent LSTM cells instead of sampled output in the early stages of training. This is useful to stabilize the training of trajectory prediction since the prediction depends on the last output which typically explodes to meaningless values in early, untrained stages. We set an initial epoch of 50 as the teaching period such that the possibility of using the ground-truth label is $1 - \text{epoch}/50$. This gradually decreasing possibility becomes 0 at epoch 50, after which ground-truth is not used.

For evaluation, linear extrapolation along with naive LSTM are selected as baselines. In the case of extrapolation, x and y coordinates of the previous 8 steps are extrapolated through time and the next 12 consecutive steps are recorded. The naive LSTM used the same state variables (x, y, v_x, v_y) and the same output structure, but only contained the LSTM encoder and MLP de-

coder (i.e. was missing an attention core). This implies that no effects of interactions with others are considered by the naive LSTM. Averaged displacement error (ADE) is calculated by taking the average of Euclidean distances from ground-truth to predicted coordinates for all 12 steps. Final displacement error (FDE) only takes the averages of the final (12th step) error.

Chimney swift flock trajectory The original paper [5] aimed to focus on collective behavior during the landing sequence of a chimney swift (CS) flock. The flock data contains 30 min of observed CS trajectories at 30 frames (=f) per second, with approximately 100,000 unique trajectories and a maximum of 1,848 birds at one instance. The reason that the number of unique trajectories greatly exceeds the size of the flock is because many of the trajectories from the same birds are treated separately if (1) they escape from the sight of the cameras and later re-enter, or (2) the birds are occluded by other birds thus introducing ambiguity. The whole dataset can be divided into three parts: (1) an initial stage where the birds are starting to gather, (2) an intermediate stage where the flock forms with birds showing collective spinning, and (3) the final stage of landing on the chimney. In this study, we used the first portion (file A) of the data since we are interested in general bird flocking rather than a specific landing sequence (file C), and the second part (file B) contained more than 3000 unique birds in a 5 s instance, generally exceeding the memory capacity of a GPU (12 GB) even for the case of a single data per batch. Among 30 min (= 54000 f) of trajectory data, we employed around 10 min (= 18000 f) of frames and constructed a dataset with $30\text{f} * 10 = 300\text{f}$ each, 20f apart from each other. Although a single datapoint spans an overlapped time range with other data, we strictly split the training and test dataset to remove any possibility of data contamination. Exact details about the dataset and statistics can be found in [5].

In the CS case, there is a problem with unbalanced labels since not every sample has full 10-step (10 s) trajectories, as mentioned in the main manuscript. Thus, we checked the number of agents present at certain steps and calculated a weighted loss to strengthen the effect of cases with fewer birds (typically, the case of a higher time step has a smaller number of constituents since many trajectories end early).

AgentNet for CS was trained with 530 sets of bird trajectories that spanned 300 frames each, split into 10 time steps. Since every trajectory starts and ends at different time steps, we take an average of bird displacement error where its starting point is regarded as time step 0. For instance, if the trajectory of bird 1 spans from time step 0 to 3 and the one of bird 2 spans from 4 to 6, the error calculated at bird 1, step 2 and bird 2, step 6 will be treated the same as the displacement error at the time step *2* since both steps are 2nd steps to their starting point. Also, since there were trajectories only lasting 2 time steps ($= 1.0$s), we cannot apply linear regression for those trajectories. Instead, we linearly extrapolated the trajectories by employing the given velocity at the first time step. Also, we intentionally scaled the input data by multiplying 0.1 for every variable to stabilize the training while preserving the relative difference of variable magnitude.

2 Supplementary Note 1 : Inspection scheme of AgentNet for CS

As the author of the original paper noted [5], CS data contains a lot of short trajectories due to frequent occlusion and limitation of the camera viewing angle. An exemplary snapshot of typical trajectory data is drawn in Fig. S1, where all of the trajectories are not aligned in time and have dif-

ferent lengths. This form of unstructured data can be expressed as a dynamic graph that precludes the naive application of a graph neural network (GNN) [6] with LSTM [7] states since the neighbor of each node changes dynamically and each node has a different starting time. Hence, we need an inspection method to manually check the hidden state at each time step and update each node's status to the appropriate form.

Figure S1 describes all three possible cases that our inspection scheme needs to deal with. Case A is an ordinary case with a continued state, so we can simply hand over the previously updated hidden state to the next iteration. Case B means that the agent, which existed at the last time step ($t = t_0 - 1$), disappeared and should not be considered as a valid neighbor anymore. Our inspection method excludes such data and creates a mask for the attention matrix to ensure that the attention between a valid agent and non-existent agent should be strictly zero at any time. Finally, case C indicates a newly entered agent whose hidden states should be initialized before it starts its chain of hidden states.

In practice, input data is created from raw data in the form of **[time step, number of total agents, number of state variables]**. Note that we use the number of *total* agents for every time step, which is not always the same as the number of agents at the specific time. For instance, in Fig. S1, there are 7 birds present in the total scene, but only 6 birds are present at $t = t_0$. Instead of changing the data size every time step, we set every dimension to the maximum agent number (in this case, 7) and fill the currently non-existing birds' state variables with dummy values (we used zeros). With such padding, we can create a batched data set that enables parallel calculations to

speed up the GPU deep learning. But we have to carefully mask them out properly at each forward pass to ensure that none of these dummy data affects the results because we keep calculating and updating these dummy data as well as the real agents' data.

Our inspection algorithm creates two masks with length of total agents, which is called 'Now mask' m_{now} and 'New mask' m_{new}. Each mask checks the existence of agents at the current($t = t_0$) and next time step ($t = t_0 + 1$), and assigns 1 if the corresponding condition is satisfied and 0 otherwise. 'Current mask' assigns 1 if the agent is present at t_0. 'New mask' assigns 1 if the agent is not present at t_0 but appears in $t_0 + 1$. Inspection method employs these three masks to control hidden state updates, attention calculations, and state variables updates.

At the start of every iteration, the algorithm checks new agents that need to be initialized. Let us denote the hidden states of agents from time t as h^t and state input data as x^t. Then, the hidden state update can be expressed as

$$h^{t+1} = h^t \odot (1 - m_{new}) + I(x^t) \odot m_{new} \tag{3}$$

where $I(x)$ indicates the hidden state initialization module consisting of neural layers, and $\odot$ means element-wise multiplication. Then, after the attention calculation, the algorithm casts a two-dimensional attention mask m_{att} on raw attention matrix α^q_{raw} to exclude all of the attention values between real and dummy agents as follows:

$$m_{att,ij} = \begin{cases} 1 & \text{if } a_i \text{ and } a_j \text{ are both real agent} \\ 0 & \text{if otherwise} \end{cases} \quad (4)$$

and $\alpha^q = \alpha^q_{raw} \odot m_{att}$. We can construct m_{att} from the 'Now mask' by repeating row vector mask m_{now} for the column dimension to make expanded matrix mask M_{now}, with $m_{att} = M_{now} \times M^T_{now}$.

3 Supplementary Note 2 : Advantages of Neural Attention

Neural attention needs greater number of parameters to optimize, but showed greater performance on predicting complex agent compared to existing attention mechanisms. Here, we compare the performance of AgentNet for Vicsek Model (VM) with different attention mechanisms to calculate attention between ith and jth agents, α_{ij}. We employed used transformer [8] architecture for all three mechanisms which seprates key k, query q and value v. Additive mechanism employs three $d \times d$ weight matrices w_1, w_2, w_3 to compute attention weight as $w_3(\sigma(w_1 k_i, w_2 q_j))$, where d is a dimension for output vector, σ is a nonlinear activation function, k_i is a key for ith agent and q_j is a query for jth agent. Multiplicative mechanism calculates attention weight with innter product as $k_i \cdot q_j$ with some normalization constant, and boradly used to handle sequential data such as a natural language.

In constrast, neural attention uses multiple MLP layers A_θ, parametrized by θ, to compute attention.

$$\alpha_{ij} = A_\theta(k_i||q_j||\mathbf{u})$$

Here, $||$ indicates the concatenation of two vectors and u is the vector of global variables. Note that the additive mechanism is technically the same as a single layer of perceptrons. Table S1 shows the results of AgentNet for VM that employs different attention mechanisms. (In this case and following demonstration, the system has 100 agents compared to 300 agents of the main manuscript version. This results in slightly lower NLL value, -1.013, compared to the one reported in the manuscript, -0.856 since the system with lower density is easier to predict.) We observed that both additive and multiplicative attention cannot fully capture the nonlinear and complex interaction range of complex systems as neural attention does.

One possible reason for the outperformance of neural attention is that the interaction range of a complex system is far more nonlinear and complicated than conventional attention mechanisms can handle, which are relatively under-parametrized for such interaction ranges. This assumption is further supported if the performance gap decreases as the interaction boundary becomes more linear. We experimented with a *smoothed* version VM (Fig. S2A), where interaction strength is defined as a sigmoid function $s(x) = 1/(1 + \exp[b(x - a)])$. Figure S2B shows the performances of VAIN (exponential-based attention from [9]), GAT (conventional multiplicative transformer), and AgentNet (neural attention) with different b when $a = 1$. Note that the original interaction boundary coincides with the $b \to \infty$ case. Results illustrate the difference between the scales of the two mechanisms for b, which underpins the aforementioned nonlinearity hypothesis.

4 Supplementary Discussion 1 : AOUP attention analysis

The variable-wise attention in AgentNet is wrapped by the sigmoid function, where its output is equal to or greater than 0, as seen in Fig. S3A. Hence, relative positional information is vital to convert the magnitude into actual force with a sign, which can be directly applied to change the velocity. For instance, force should be $+x$ direction if the target agent i is in $(1, 0)$ and the neighbor agent j is in $(0, 0)$, but it changes to $-x$ direction if the neighbor agent moves to $(2, 0)$, although the magnitude (and thus the attention value) would be the same. It is possible to regain this directional information if the value vector v_j, corresponding to a leftover function, contains the positional data of the jth agent; the position information of the ith agent can be delivered by h_{self}.

Figure S3 verifies that value vector v preserves the positional information of the input data. We gathered data from 100 test samples of AOUP, and performed principal component analysis (PCA) to 16-dimensional vector v from the encoder. Figure S3B shows that the top four principal components (PCs) stand out in terms of explained variance, compared to the PCs with lower ranks. We found that these four PCs have an interesting nonlinear relationship with two coordinates x and y from input data. Figure S3C shows that these four PCs have strong sinusoidal relationships with x and y, giving clear evidence of information preservation. We note that this specific non-linear transformation is not special, and there could be various other possible means of preserving positional information. (For instance, the simplest way to convey positional information would be to directly preserve the coordinate data from the input without any nonlinear transformation.) In this particular case, we can assume that the overall function f could successfully retrieve the

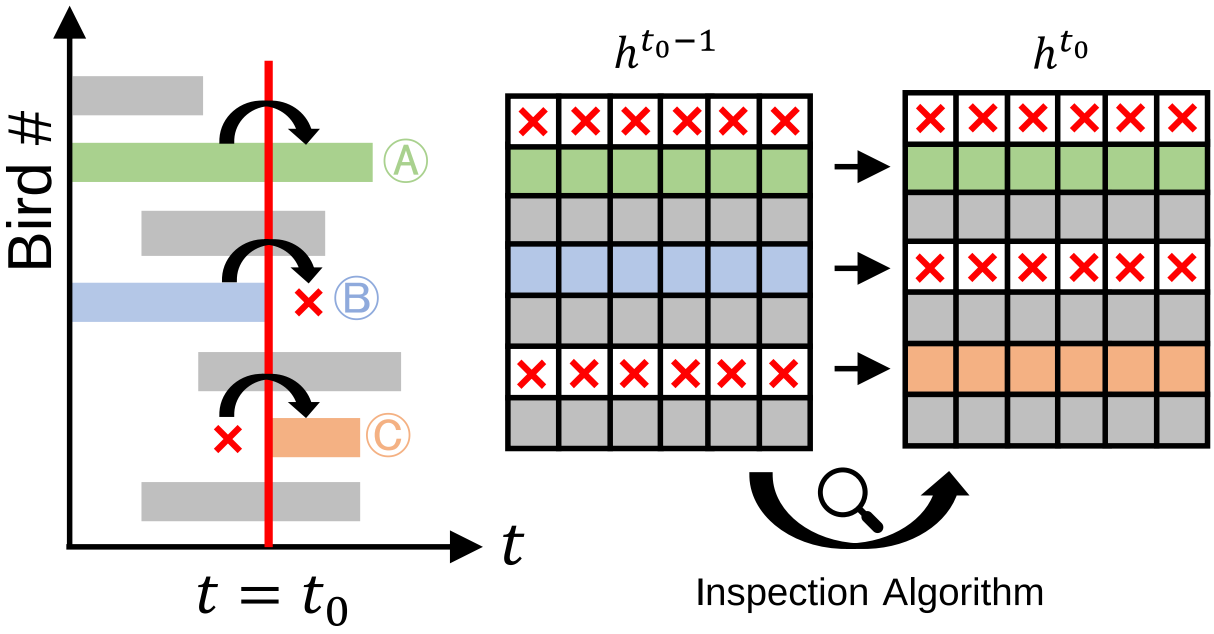

Figure S1: Trajectory data schema of the chimney swift flock [5]. (left) In each time step, each agent (bird) could take one of three states: **(A)** it continues to exist, **(B)** it disappears, or **(C)** it newly enters the scene. (right) The hidden states of the agents for LSTM change by the inspection algorithm, correctly removing vanished agents and introducing new agents with initialized hidden states. Red crosses indicate dummy variables (zeros) for padding.

original coordinate from these sinusoidal functions and ultimately obtain the sign of the relative position by comparing it with the target ith agent's position.

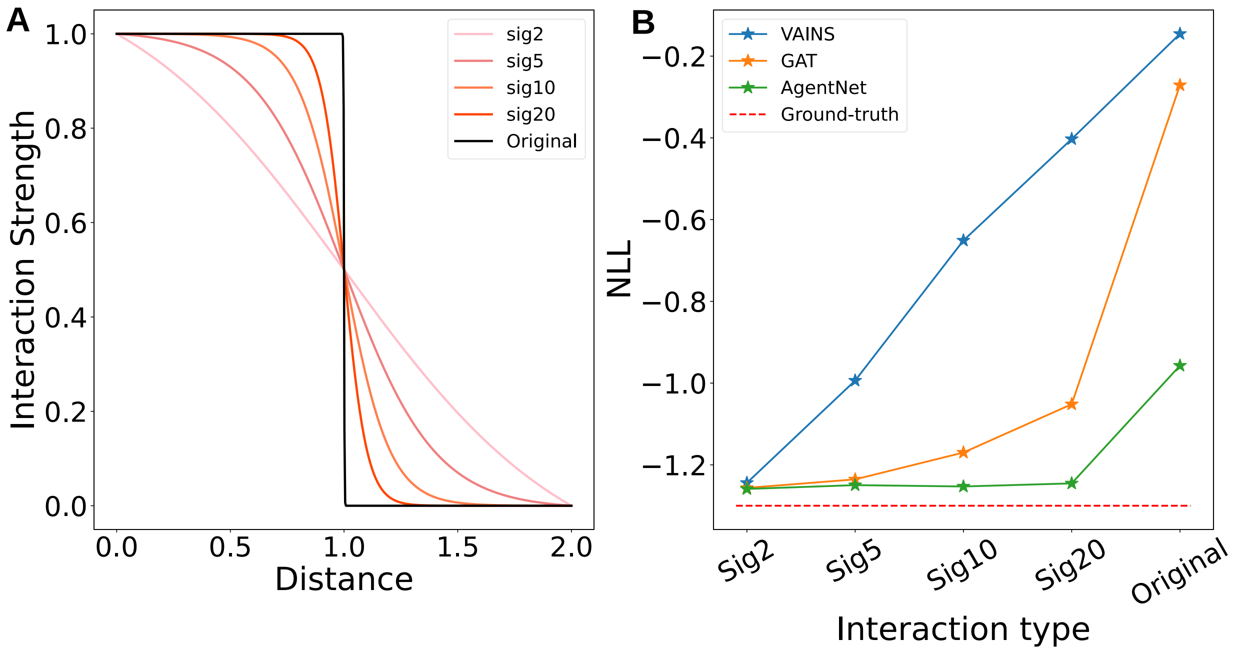

Figure S2: **(A)** Functions of a *smoothed* version of interaction range from sigmoid models with various smoothing parameter b. **(B)** Performance comparison of VAINS, GAT, and AgentNet compared to NLL loss from groundtruth.

5 Supplementary Figure 1: Figure S1

6 Supplementary Figure 2: Figure S2

7 Supplementary Figure 3: Figure S3

8 Supplementary Table 1: Table S1

9 Supplementary References

1. Gardner, M. Mathematical games. *Scientific American* **222**, 132–140 (1970).

2. Vicsek, T., Czirók, A., Ben-Jacob, E., Cohen, I. & Shochet, O. Novel type of phase transition in a system of self-driven particles. *Physical Review Letters* **75**, 1226 (1995).

3. Toral, R. & Colet, P. *Stochastic numerical methods: an introduction for students and scientists* (John Wiley & Sons, 2014).

4. Williams, R. J. & Zipser, D. A learning algorithm for continually running fully recurrent neural networks. *Neural computation* **1**, 270–280 (1989).

5. Evangelista, D. J., Ray, D. D., Raja, S. K. & Hedrick, T. L. Three-dimensional trajectories and network analyses of group behaviour within chimney swift flocks during approaches to the roost. *Proceedings of the Royal Society B: Biological Sciences* **284**, 20162602 (2017).

6. Kipf, T. N. & Welling, M. Semi-supervised classification with graph convolutional networks. *arXiv preprint arXiv:1609.02907* (2016).

7. Hochreiter, S. & Schmidhuber, J. Long short-term memory. *Neural computation* **9**, 1735–1780 (1997).

8. Vaswani, A. *et al.* Attention is all you need. In *Advances in neural information processing systems*, 5998–6008 (2017).

9. Hoshen, Y. Vain: Attentional multi-agent predictive modeling. In *Advances in Neural Information Processing Systems*, 2701–2711 (2017).

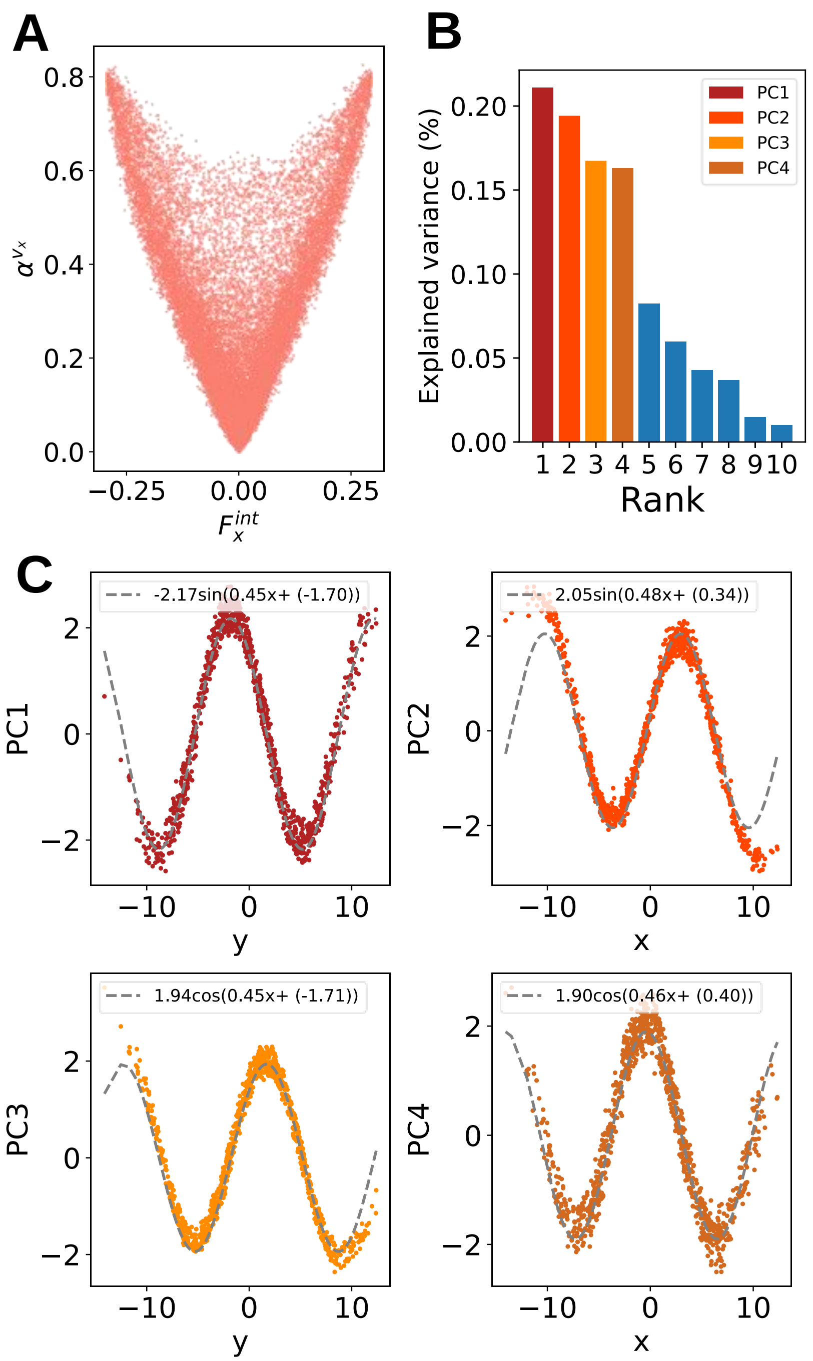

Figure S3: PCA result of value vectors v from AgentNet for AOUP. **(A)** x-directional force $F^{int,x}$ abd attention for v_x. Note that attention learns magnitude not the sign, due ot its non-negative constraint. **(B)** Explained variance of each principal component sorted by rank, which shows four clearly distinguished components. **(C)** Each principal component and corresponding x or y coordinate data.

Table S1: Comparison between different attention mechanism for AgentNet for VM. Neural attention showed best result among other conventional attention mechanisms.

Attention	Train loss (NLL)	Test Loss (NLL)
Additive attention	-0.694	-0.672
Muliplicative attention	-0.778	-0.743
Neural attention	**-1.013**	**-1.021**
